# The glial sodium-potassium-2-chloride cotransporter is required for synaptic transmission in the *Drosophila* visual system

**DOI:** 10.1038/s41598-019-38850-x

**Published:** 2019-02-21

**Authors:** Drew Stenesen, Andrew T. Moehlman, Jeffrey N. Schellinger, Aylin R. Rodan, Helmut Krämer

**Affiliations:** 1 Department of Neuroscience, University of Texas Southwestern Medical Center, Dallas, TX 75390 USA; 2 Department of Internal Medicine, Division of Nephrology, University of Texas Southwestern Medical Center, Dallas, TX 75390 USA; 3 Department of Cell Biology, University of Texas Southwestern Medical Center, Dallas, TX 75390 USA; 4 Department of Internal Medicine, Division of Nephrology and Hypertension and Molecular Medicine Program, University of Utah, Salt Lake City, UT 84112 USA; 5 Medical Service, Veterans Affairs Salt Lake City Health Care System, Salt Lake City, Utah USA; 6 Present Address: Biology Department, University of Dallas, Irving, TX 75062 USA

## Abstract

The *Drosophila Ncc69* gene encodes a Na^+^-K^+^-2Cl^−^-cotransporter (NKCC) that is critical for regulating intra- and extracellular ionic conditions in different tissues. Here, we show that the Ncc69 transporter is necessary for fly vision and that its expression is required non-autonomously in glia to maintain visual synaptic transmission. Flies mutant for *Ncc69* exhibit normal photoreceptor depolarization in response to a light pulse but lack the ON and OFF-transients characteristic of postsynaptic responses of lamina neurons, indicating a failure in synaptic transmission. We also find that synaptic transmission requires the Ncc69 regulatory kinases WNK and Fray in glia. The ERG phenotype is associated with a defect in the recycling of the histamine neurotransmitter. *Ncc69* mutants exhibit higher levels of the transport metabolite carcinine in lamina cartridges, with its accumulation most intense in the extracellular space. Our work reveals a novel role of glial NKCC transporters in synaptic transmission, possibly through regulating extracellular ionic conditions.

## Introduction

Neuronal activity is defined by ionic flux. Coordinated control of ion gradients is therefore critical for brain function, and failure of such control leads to neurological diseases, including seizures, schizophrenia, and neuropathic pain^1–3^. Glia isolate neurons from surrounding chemistries^4^ and buffer the sudden ionic flux that accompanies neurotransmission^5,6^. Important contributors to this homeostatic regulation are the K^+^-Cl^−^ cotransporters (KCCs) and Na^+^-K^+^-2Cl^−^ cotransporters (NKCCs)^2,7,8^. These cation-chloride cotransporters (CCCs) function as secondary active symporters that utilize Na^+^ or K^+^ gradients to drive Cl^−^ movement. Under the typical ionic conditions generated by the Na^+^/K^+^-ATPase, the NKCCs are positioned for ionic import while KCCs drive export^3,9^.

Accordingly, NKCCs and KCCs are subject to functionally opposing posttranslational modifications. The family of with-no-lysine(K) or WNK kinases phosphorylate the STE20/SPS-related proline/alanine-rich kinase (SPAK) and Oxidative stress response 1 (OSR1) kinases, thereby activating them. Activated SPAK/OSR1, or Fray in *Drosophila*^10^, phosphorylate NKCCs and KCCs, which leads to their activation and deactivation respectively^1^. The regulatory function of this module is conserved in *Drosophila*: *WNK* and the SPAK/OSR1 homolog *Fray* regulate the NKCC1 homolog Ncc69^10–15^.

**Figure 1:**
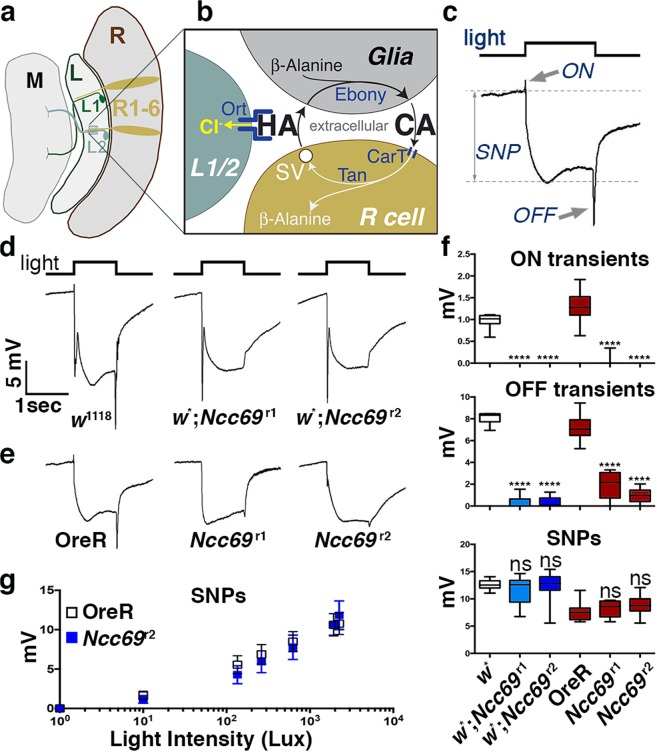
Loss of *Ncc69* blocks visual neurotransmission. (**a**) Schematic drawing of the *Drosophila* visual system highlighting the Retina (R) that houses the photoreceptor cells (R1-6), the Lamina (L) with L1 and L2 as the main postsynaptic neurons, and the Medulla (M). (**b**) Schematic of photoreceptor cell synapses containing the histamine-gated channel Ort, and the histamine (HA) recycling pathway, including Ebony which catalyzes the reaction of histamine and β-alanine to carcinine (CA) in glia^50^, CarT which transports carcinine into photoreceptor axons^25,35,36^ and Tan which regenerates histamine by carcinine hydrolysis^61^. (**c**) Example of a wild-type ERG recording pointing to its ON- and OFF-transient and SNP components. (**d,e**) ERGs recorded from wild-type control and *Ncc69* mutants in either a *w*^*−*^ (**d**) or *w*^+^ (**e**) background. In both backgrounds, loss of Ncc69 diminished or abolished ON and OFF transients. (**f**) ON transients, OFF transients, and SNPs were quantified from three replicate experiments including at least 45 traces from 15 flies. Graphs report median, upper and lower quartiles (box) and minimum and maximum values (whiskers). ns, not significant; ****p < 0.0001 compared to the corresponding *w*^1118^ or OreR controls. (**g**) Average SNPs from *Ncc69* flies measured over a range of light intensities were compared to responses from wild type (Means from three replicate experiments including at least 45 traces from 15 flies). Error bars represent standard deviation. Paired values were not significantly different by Student’s t-test.

In the *Drosophila* peripheral nervous system, glial CCCs control the ionic environment in larval nerves^13,16^. Nevertheless, loss of *Ncc69* function had little impact on neuronal function, except a slightly increased seizure susceptibility^16^. Here, we explore the role of the WNK-Fray-Ncc69 module in the context of the visual system (Fig. 1a). *Drosophila* photoreceptors use histamine as neurotransmitter^17,18^. Histamine synthesis in photoreceptor neurons is insufficient to sustain their high rate of synaptic release, and therefore histamine recycling is critical for normal vision^19^. Upon light activation of photoreceptors, synaptic release of histamine opens histamine-gated chloride channels in postsynaptic lamina neurons, causing their hyperpolarization^20^. These synapses are organized in cartridges^21^, each containing six photoreceptor axons and the dendrites of postsynaptic lamina neurons. Individual cartridges are ensheathed by a set of three astrocyte-like glia that recycle histamine neurotransmitter (Fig. 1b) and isolate individual cartridges^19,22,23^. Our data indicate that the module consisting of the sodium potassium chloride symporter, Ncc69, and its regulatory kinase cascade is necessary in glia for *Drosophila* vision.

## Results

### *Ncc69* is required for photoreceptor neurotransmission

To examine a role for Ncc69 in visual neurotransmission, we used electroretinograms (ERGs) to measure the sustained negative potential (SNP) of photoreceptor depolarization in response to a light pulse and the postsynaptic responses from lamina neurons, known as ON- and OFF transients, that correspond to the initiation and termination of a light pulse, respectively (Fig. 1c). ERG recordings of flies homozygous for the two strong loss-of-function alleles *Ncc69*^r1^ and *Ncc69*^r2^ ^12,13^ revealed severely reduced ON and OFF transients, indicating a disruption in neurotransmission (Fig. 1d–f). Disruption of neurotransmission was independent of the function of *white* (*w*), which is required for eye pigmentation and is known to affect various aspects of *Drosophila* vision^24^. Of note, compared to controls, none of these genotypes displayed significantly reduced SNPs (Fig. 1f), irrespective of intensity of the light pulse (Fig. 1g), indicating that loss of *Ncc69* did not affect the ability of photoreceptor neurons to depolarize in response to light.

**Figure 2:**
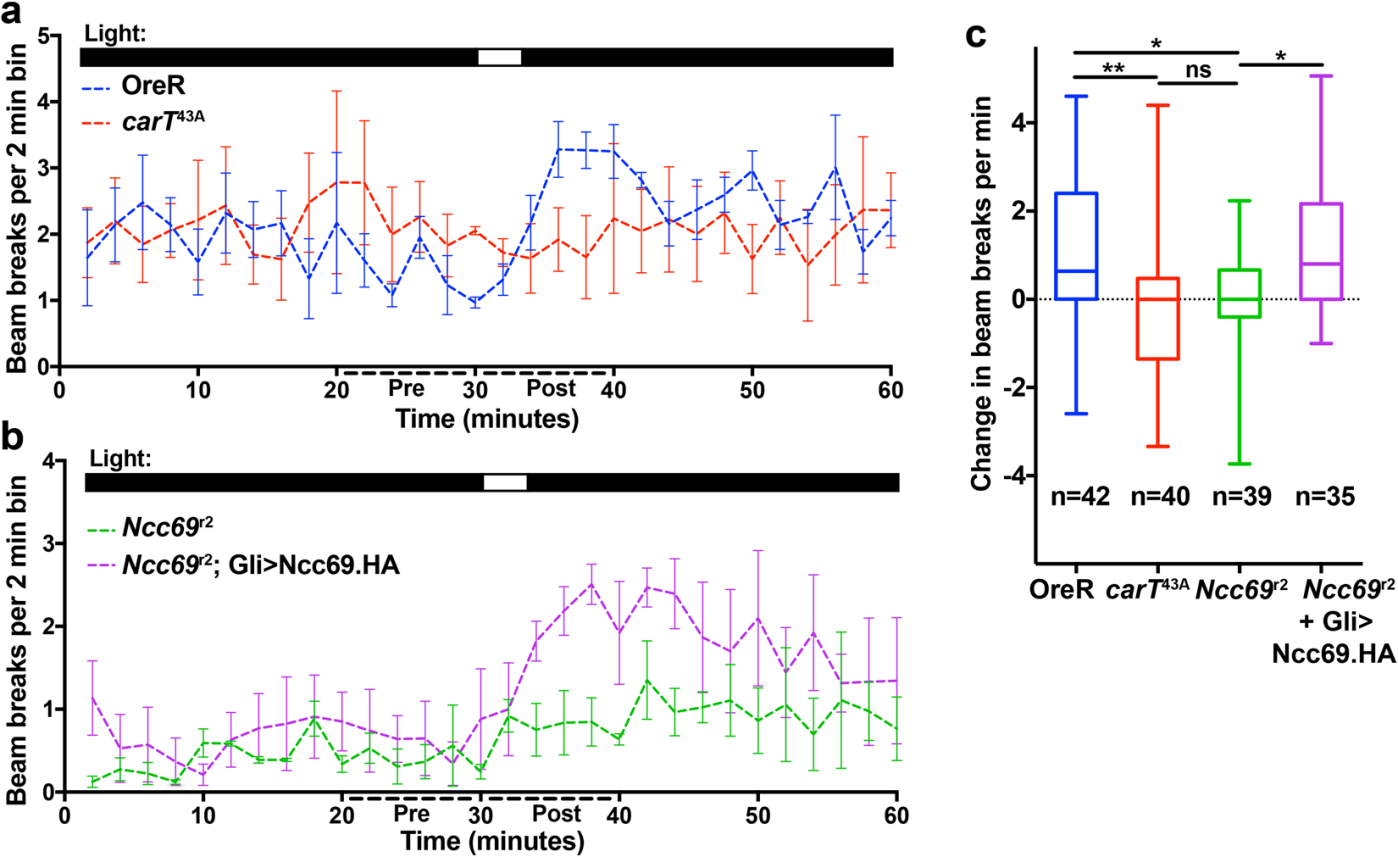
Loss of light-startle response in *Ncc69*^*r2*^ mutants is rescued by glial Ncc69 expression. (**a,b**) Actogram plots of OreR (positive control) and histamine-recycling defective *carT*^43A^ flies (negative control) (A) or *Ncc69*^r2^ and *Ncc69*^*r2*^;*Gli*-Gal4 > UAS-Ncc69.HA flies (**b**) for 30 min prior to and 25 min following a 5-min light pulse. Light pulse is indicated by upper bars. All flies were red-eyed. Error bars indicate S.E.M. (**c**) Quantification of change in beam breaks per min for the 10 min intervals before and after the onset of the light pulse in each experiment for the indicated genotypes. Plots show Box & Whisker plots representing all the flies from three technical replicates. Graphs report median, upper and lower quartiles (box) and minimum and maximum values (whiskers). Biological n for each sample is indicated. *p < 0.05; **p < 0.01.

To test the behavioral consequences of loss of *Ncc69* function, we employed an automated ‘startle-response’ assay. Once adapted to the dark for 2 hours, wild-type flies, but not histamine-recycling defective *carT*^43A^ mutants^25^, responded to a short light pulse with elevated motor activity (Fig. 2a,c). Similarly, loss of *Ncc69* function eliminated the light-evoked startle response (Fig. 2b,c). Together with the ERG findings, these data indicate that Ncc69 function is required for normal visual responses.

**Figure 3:**
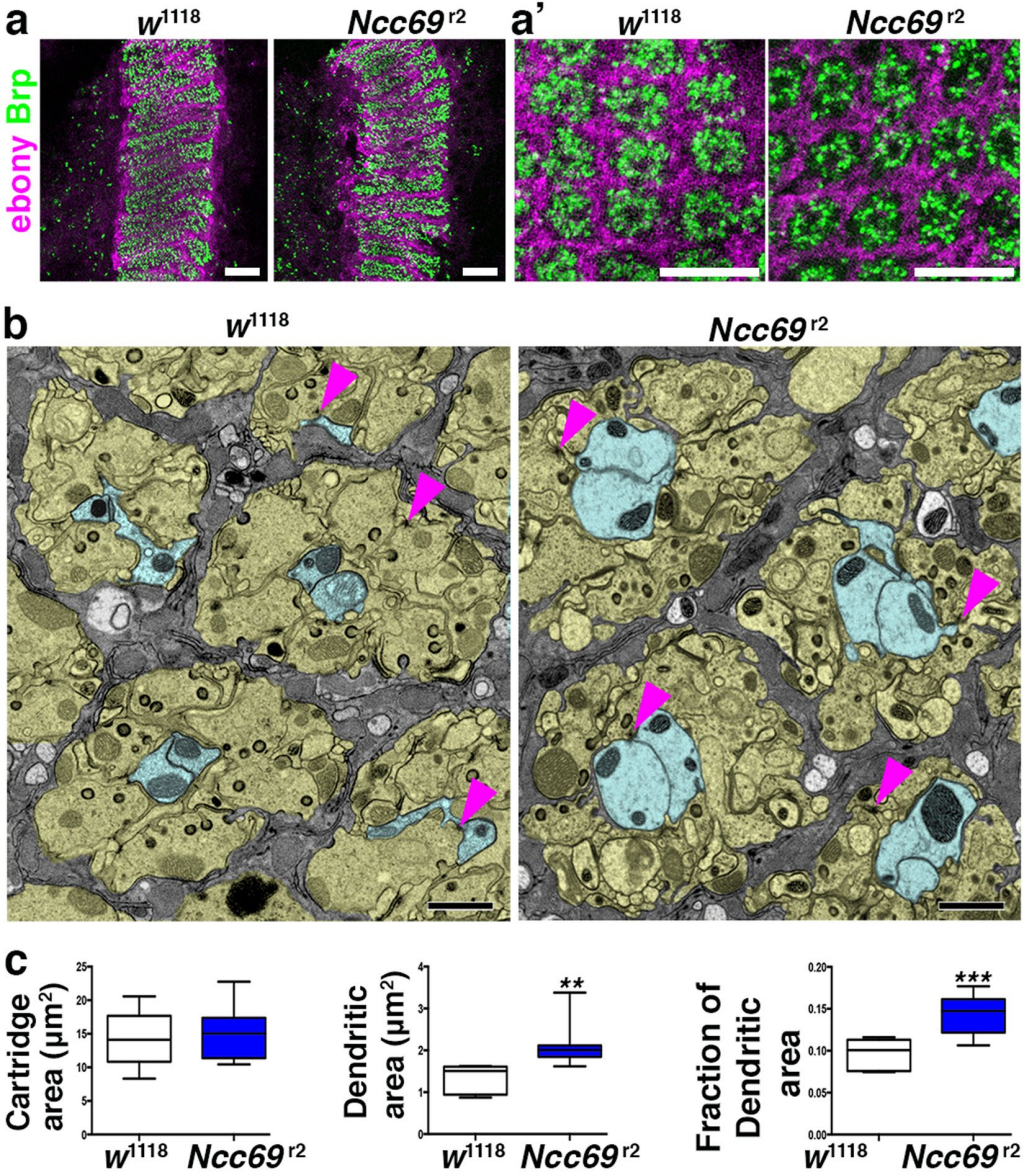
Loss of *Ncc69* alters lamina neuron morphology. (**a**) Confocal images of longitudinal (**a**) and cross-sections (a’) through lamina cartridges of control (*w*^1118^) and *Ncc6*9^r2^ flies stained for Bruchpilot and Ebony, showing intact structure of photoreceptors and glia. (**b**) Electron micrographs of control (*w*^1118^) and *Ncc69*^r2^ mutant laminae displaying glial cells (grey) surrounding individual cartridges containing photoreceptor axons (tan) and postsynaptic L1/2 dendrites (blue). Note that *w*^1118^ was used as control, as *Ncc69*^r2^ mutants were in a *w* background. Arrowheads point to T-bars revealing photoreceptor synapses. (**c**) Quantification of areas for lamina cartridges and L1/2 dendrites and the fraction of L1/2 dendrite areas per cartridge within the lamina neuropil. Student’s t-test: **p < 0.01, ***p < 0.001. Scale Bars: 10 µm in A, 1 µm in B.

To further assess the health of the visual circuit in *Ncc69* mutants, we stained wild-type and *Ncc69*^*r2*^ cryosections for the pre-synaptic marker Bruchpilot. Within the lamina region, marked by co-staining for the glial-specific enzyme Ebony, synaptic density and distribution were similar in flies lacking *Ncc69* compared to wild-type controls (Fig. 3a). In addition, electron micrographs of the lamina region (Fig. 3b) demonstrated that overall cartridge organization was unaltered, including T-bars, capitate projections, and synaptic vesicles. Interestingly, cross-sectional areas of L1 and L2 dendrites were increased (Fig. 3c). This increase is reminiscent of the role of *Ncc69* in volume regulation in larval abdominal nerves. In larval peripheral nerves, however, the major volume changes affected the extracellular fluid between glia and axons^13^. Such extracellular fluid accumulation was not detected in the visual system, suggesting that different physiological consequences result from loss of *Ncc69* function, depending on cellular context.

### Ncc69 is expressed in glia

**Figure 4:**
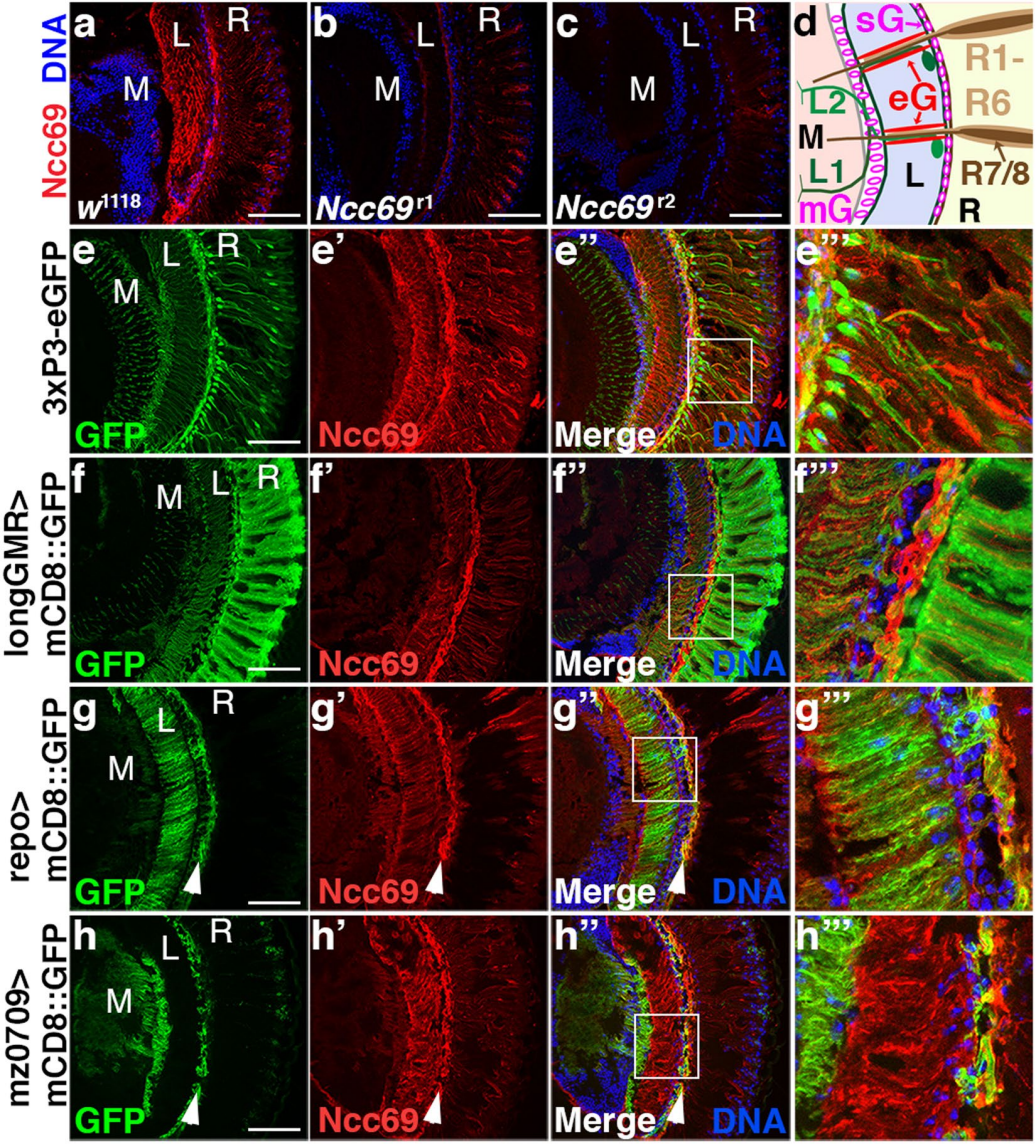
Ncc69 is expressed in glia of the visual system. (**a**–**c**) Micrographs of cryo-sections from *w*^1118^ (**a**), *Ncc69*^r1^ (**b**) or *Ncc69*^r2^ (**c**) fly heads stained for Ncc69 (red) and DNA (blue). (**d**) Schematic drawing of the *Drosophila* visual system highlighting the Retina (R) with photoreceptor cells R1-6 projecting to the lamina (L) and R7/8 projecting to the medulla (M). L1 and L2 are the main postsynaptic neurons in the lamina; their dendrites and photoreceptor axons form cartridges that are ensheathed by epithelial glia (eG). The lamina is separated from the retina by satellite glia (sG) and from the medulla by marginal glia (mG). (**e**–**h**) Ncc69 immunohistochemistry (red) on cryo-sections from flies expressing GFP (green) in photoreceptors (E, 3xP3-eGFP), or UAS-mCD8::GFP (green) in photoreceptors (**f**, longGMR-Gal4), glia (**g**, *repo*-Gal4) or satellite glia (**h**, mz0709-Gal4). Ncc69 antibody staining is present adjacent to the photoreceptor neurons, overlapping with lamina glia. Arrowheads point to satellite glia in the distal lamina. Scale bars: 50 µm. M: medulla, L: Lamina, R: Retina.

In larvae, Ncc69 expression is highest in glia^13^. Cryosections of adult wild-type brains showed that Ncc69 was highly expressed in the lamina (Fig. 4a); specificity of antibody staining was confirmed by reduced Ncc69 levels in *Ncc69*^*r1*^ and its absence in *Ncc69*^*r2*^ mutants (Fig. 4b,c). *Ncc69*^*r2*^ mutants were used for all further analysis. In the retina or lamina, Ncc69 exhibited only little co-localization with GFP expressed under the control of the photoreceptor-specific 3xPax3 promoter (Fig. 4e). To further examine localization of Ncc69 within specific cell types, we utilized the Gal4/UAS system to express a membrane-bound mCD8::GFP in photoreceptor neurons via the longGMR-Gal4 driver (Fig. 4f) or in glia via the *repo*-GAL4 driver (Fig. 4g). Ncc69 antibody staining largely overlapped with lamina glia. Interestingly, compared to the epithelial glia present throughout the lamina^26^, Ncc69 was enriched in the more distal regions of the lamina which house satellite glia (Fig. 4d). This observation was confirmed by Ncc69 antibody staining of brains expressing mCD8::GFP under control of mz0709-Gal4 (Fig. 4h), a driver expressed in marginal and satellite glia^27,28^. Taken together these data indicate that Ncc69 is expressed at high levels in multiple glial sub-types within the lamina.

### Glial-specific requirement for *Ncc69*

**Figure 5:**
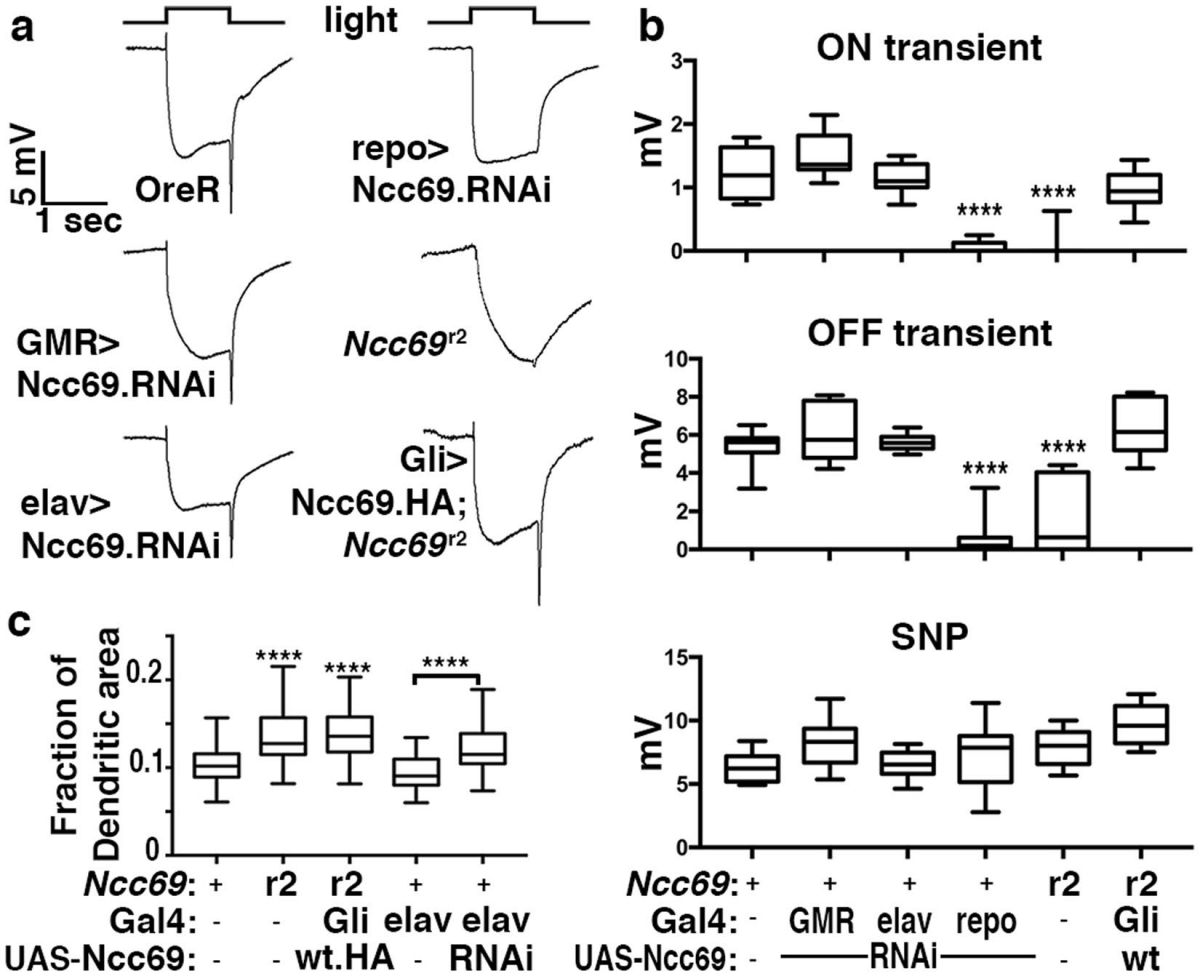
Ncc69 is required in glia but not neurons for normal visual responses. (**a**) ERGs recorded from flies expressing a UAS-Ncc69 RNAi transgene (VDRC 106499) targeting *Ncc69* under the control of the indicated Gal4 driver expressed either pan-neuronally (*elav*-Gal4), in photoreceptors (GMR-Gal4) or in glia (*repo*-Gal4). ERGs were also recorded from *Ncc69*^r2^ flies expressing an HA-tagged UAS-Ncc69 transgene driven by the glial *Gli*-Gal4 driver (compare to *Ncc69*^r2^). (**b**) Quantification of ON transients, OFF transients, and sustained negative photoreceptor potentials (SNPs) of genotypes in panel H averaged from three replicate experiments including at least 45 traces from 15 flies. (**c**) Quantification of the fraction of L1/2 dendrite areas per cartridge from light micrographs of lamina neuropil from *w*^1118^ controls, *Ncc69*^r2^, *Ncc69*^r2^ functionally rescued by a *Gli*-Gal4-driven UAS-Ncc69 transgene, or flies expressing an *elav*-Gal4-driven UAS-Ncc69 RNAi transgene (VDRC 106499). Graphs report median, upper and lower quartiles (box) and minimum and maximum values (whiskers). ****p < 0.0001 compared to wild-type control.

To identify which cell types require *Ncc69* for normal neurotransmission, we performed ERG analysis on flies expressing an RNAi transgene targeting the *Ncc69* transcript. Consistent with the localization studies, ERG components were unaltered when *Ncc69-RNAi* was expressed in photoreceptor neurons via GMR-Gal4 (Fig. 5a,b) or pan-neuronally via *elav*-Gal4, indicating that *Ncc69* is not necessary in photoreceptors or post-synaptic L1/2 neurons (Fig. 5a,b). By contrast, pan-glial knockdown of Ncc69 by *repo*-Gal4-driven *Ncc69-RNAi* (Supplemental Fig. 1c) caused loss of ON- and OFF transients, phenocopying *Ncc69* null alleles (Fig. 5a,b). Surprisingly, *Ncc69* knockdown using Gal4 drivers specific for epithelial glia or other subsets of glia (as indicated with arrows in Supplemental Fig. S1c) did not cause consistent loss of ON and OFF transients (Supplemental Fig. S1a,b) in contrast to the pan-glial *repo*-Gal4-driven knockdown. Because low levels of Ncc69 could still be detected after knockdown with some of these drivers, we cannot distinguish whether the remaining Ncc69 protein is sufficient in the critical cell types or whether Ncc69 expression in a subset of glia is sufficient to control ionic conditions in the extracellular milieu of the lamina.

To determine whether glial expression of Ncc69 in an otherwise *Ncc69* mutant background was sufficient to restore normal visual neurotransmission, we tested whether the *Ncc69*^r2^ phenotype could be rescued by cell type-specific expression of *Ncc69*. Glial-specific expression of *Ncc69* under control of *gliotactin* (*Gli*)-Gal4^29,30^ (Supplemental Fig. 2) was sufficient to restore ON- and OFF transient defects of *Ncc69*^r2^ mutants (Fig. 5a,b). Importantly, glial-specific Ncc69 rescue also restored the behavioral response to light startle (Fig. 2b,c). Taken together, these data indicate that *Ncc69* is necessary at least in some subset of glia, but not neurons, for proper visual neurotransmission.

As glial Ncc69 function has previously been linked to volume control^13^, we wondered whether the increased L1/L2 dendritic area (Fig. 3b,c) contributed to the loss of ON- and OFF transients. Interestingly, glial-specific expression of Ncc69 (Supplemental Fig. 2) rescued neurotransmission (Fig. 5a,b), but failed to restore the increased L1/L2 dendritic area of *Ncc69*^*r2*^ flies to wild-type levels (Fig. 5c). Furthermore, the increase in *Ncc69*^*r2*^ L1/L2 dendritic area was phenocopied by pan-neuronal expression of Ncc69-RNAi (Fig. 5c), despite normal ON- and OFF transients (Fig. 5a,b). These data functionally uncouple the L1/L2 volume changes from the loss of ON- and OFF transients and indicate that these L1/L2-specific changes are not critical for the loss of neurotransmission.

### Glial-specific knockdown of WNK and Fray kinases phenocopies *Ncc69* loss-of-function

**Figure 6:**
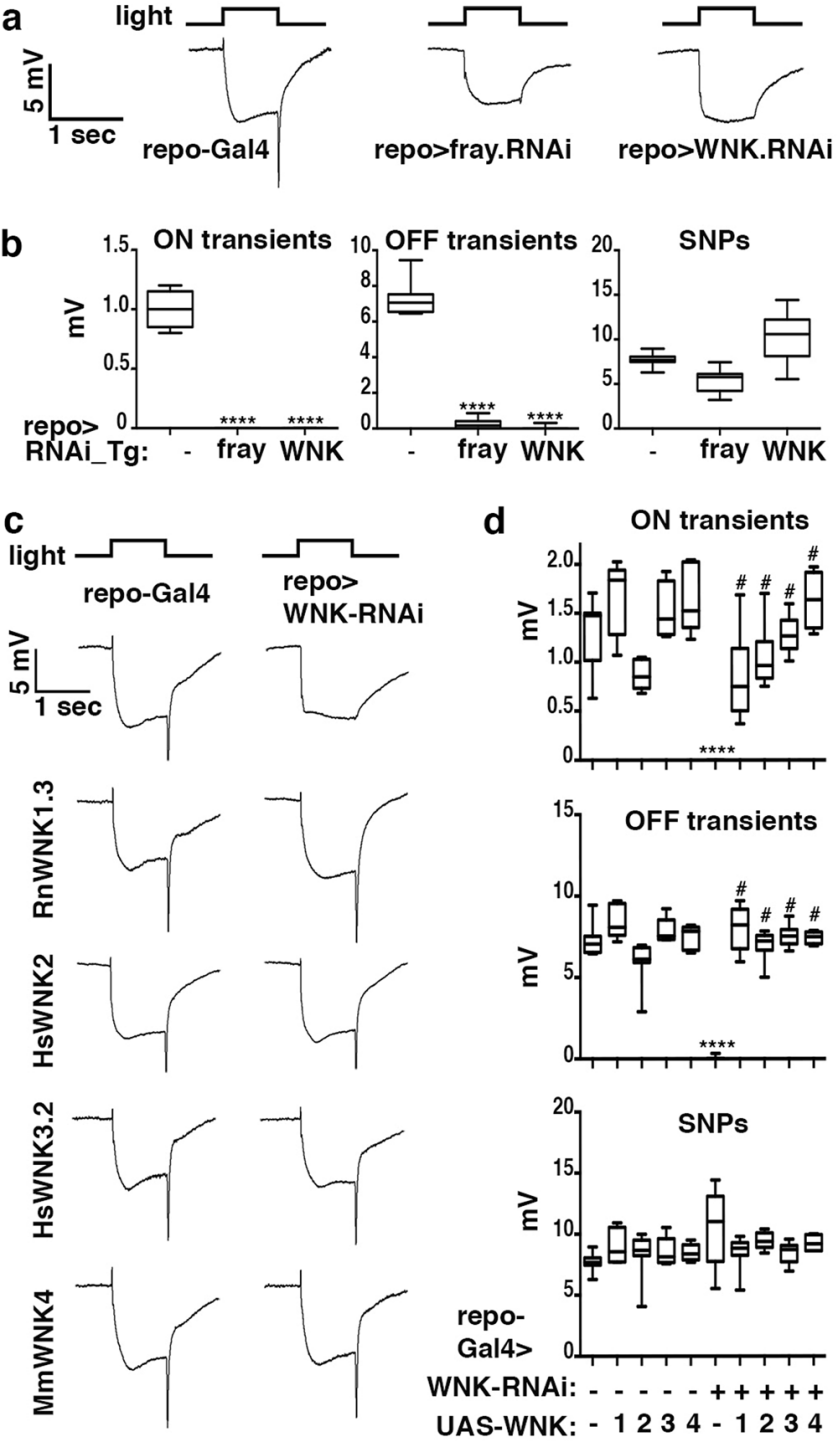
WNK and Fray kinases are required in glia but not neurons for normal visual response. (**a**) ERG recordings of flies expressing Fray or WNK RNAi constructs under control of the *repo*-Gal4 driver reveal the lack of ON and OFF transients. (**b**) Quantification of ON- and OFF-transients, and SNPs of genotypes in panel A averaged from three replicate experiments including at least 45 traces from 15 flies. (**c**) ERGs of flies with *repo*-Gal4-driven expression of either a WNK-RNAi alone or WNK-RNAi together with the indicated mammalian WNK transgenes. (**d**) Quantification of ON transients, OFF transients, and sustained negative photoreceptor potentials (SNPs) of genotypes in panel C averaged from three replicate experiments including at least 45 traces from 15 flies. Graphs (**b,d**) report upper and lower quartiles (box) and minimum and maximum values (whiskers). ns, not significant; ****p < 0.0001 compared to OreR; *repo*-Gal4 control; ^#^p < 0.0001 compared to *repo*-Gal4; UAS-WNK.RNAi.

Further support for the role of glia in Ncc69-mediated transport came from studies of the regulatory Fray and WNK kinases. Like their mammalian homologs^1^, *Drosophila* WNK kinase phosphorylates Fray^31,32^, which in turn phosphorylates Ncc69 and activates its Na^+^/K^+^/2Cl^−^ import activity^10,33^. To test whether this regulatory WNK-Fray-Ncc69 cassette is also critical in the *Drosophila* visual system, we knocked down *Fray* or *WNK* in glia using the *repo*-Gal4 driver and RNAi transgenes, because null alleles for both kinases are lethal^14,32,34^. Consistent with their known role of regulating Ncc69 in larval glia and in other tissues^10,13,33^, glial-specific knockdown of either WNK or Fray phenocopied *Ncc69* with regard to loss of ON and OFF transients (Fig. 6a,b). These data are consistent with a requirement for the WNK-Fray-Ncc69 cassette for proper neurotransmission in the *Drosophila* visual system.

### Mammalian WNKs rescue *Drosophila* neurotransmission defects

The *Drosophila* genome contains a single *WNK* homolog while mammalian genomes, including the human genome, contain four homologs. To test whether mammalian *WNKs* are functionally conserved in the context of *Drosophila* vision, we examined the ability of mammalian *WNK* homologs to rescue the ERG defects seen upon glial-specific knockdown of *Drosophila WNK*. To achieve this, we used *repo*-Gal4 to drive expression of either UAS-mammalian *WNK* alone or in combination with UAS-WNK-RNAi. Importantly, the WNK RNAi transgene targets the *Drosophila* transcript outside of the highly conserved kinase domain in a region sufficiently divergent from the mammalian *WNK* transcripts to avoid their degradation. Expression of mammalian *WNK* in an otherwise wild-type background did not interfere with ERG components, with the exception of *WNK2* overexpression, which caused a slight but significant reduction in ON- and OFF transients (Fig. 6c,d). However, when expressed in the presence of glia-specific *Drosophila WNK* knockdown, all four mammalian *WNK* homologs restored both ON- and OFF transients (Fig. 6c,d), consistent with a potential WNK-Fray-Ncc69 regulatory cassette being required in glia for visual neurotransmission.

### Carcinine accumulates in *Ncc69* mutant lamina

**Figure 7:**
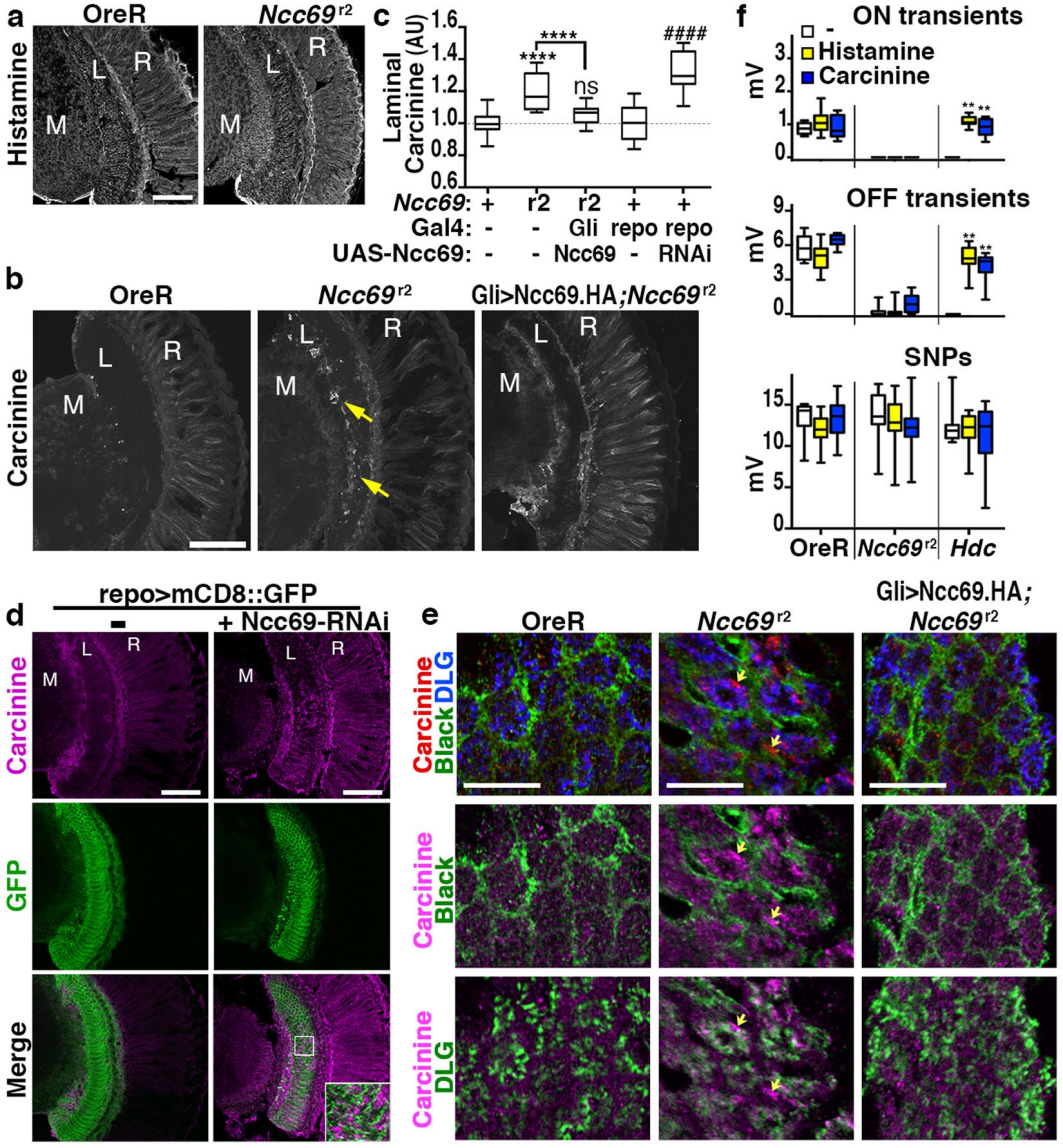
Loss of Ncc69 in glia leads to extracellular carcinine accumulation. (**a**) Micrographs show confocal sections of control and *Ncc69*^r2^ heads stained for histamine or (**b**) control, *Ncc69*^r2^, and *Ncc69*^r2^ heads expressing an UAS-Ncc69 transgene driven by *Gli*-Gal4 stained for carcinine. Carcinine accumulates in *Ncc69*^r2^ lamina (arrows). (**c**) Quantification of lamina carcinine in *OreR*, *Ncc69*^r2^, and *Ncc69*^r2^ expressing an HA-tagged UAS-Ncc69 transgene driven by the glial *Gli*-Gal4 driver as well as *repo*-Gal4 > UAS-mCD8::GFP without or with UAS-Ncc69-RNAi. ns, not significant; ****p < 0.0001 compared to *OreR* or ^####^p < 0.0001 compared to *repo*-Gal4. (**d**) Micrographs of sections stained for carcinine from fly heads expressing *repo*-Gal4-driven UAS-mCD8::GFP with or without UAS-Ncc69-RNAi. (**e**) Airyscan confocal slices of carcinine accumulation in lamina of *Ncc69*^*r2*^ mutants, compared to *OreR* or *Gli*-Gal4, UAS-Ncc69.HA*; Ncc69*^*R2*^ flies. Sections were co-labeled for the lamina epithelial glia marker Black and for DLG to label photoreceptor axons. Arrows indicate sites of strong extracellular carcinine accumulation. Scale bars: 50 µm in (**b**,**c**,**e**) or 10 µM in f. M: medulla, L: Lamina, R: Retina. (**f**) Quantification of ON- and OFF-transients, and SNPs of control, *Ncc69*^r2^, and *Hdc* mutants fed either a normal diet (−) or one supplemented with histamine or carcinine. **p < 0.0001 compared to each genotype’s normal diet condition.

One mechanism by which lamina glial cells contribute to Drosophila vision is the recycling of histamine neurotransmitter (Fig. 1b). Mutations in the enzymatic or transport components necessary for histamine recycling are known to inhibit visual neurotransmission and redistribute histamine neurotransmitter, or its transport metabolite carcinine, in the retina and lamina^24,25,35–40^. To test whether loss of *Ncc69* impacts this histamine-carcinine cycle, we performed immunofluorescence staining for these two metabolites. *Ncc69*^*r2*^ mutants displayed no detectable accumulation of histamine in retina or lamina when compared to wild-type (Fig. 7a). By contrast, carcinine levels were increased in *Ncc69*^*r2*^ lamina (Fig. 7b, arrows). Importantly, wild-type carcinine distribution was restored in *Ncc69*^*r2*^ flies expressing a UAS-Ncc69.HA transgene specifically in glial cells (Fig. 7b,c). In addition, knockdown of *Ncc69* specifically in glia, using *repo*-Gal4 to drive UAS-Ncc69-RNAi, was sufficient to increase carcinine levels (Fig. 7c,d). In order to determine the location of carcinine buildup within the lamina, we marked either photoreceptors with *3xPax3-GFP* (Supplemental Fig. S3a) or glial cells with *repo*-Gal4-driven membrane bound mCD8::GFP (Fig. 7d) or cytoplasmic tdGFP (Supplemental Fig. S3b). These cellular markers showed only a low degree of colocalization with carcinine that accumulated in the lamina upon *Ncc69* knockdown in glia (insets in Fig. 7d, Supplemental Fig. S3a,b). Furthermore, high resolution Airyscan images detected carcinine accumulations in *Ncc69*^*r2*^ lamina cartridges with the most pronounced buildups not colocalizing with either staining for Black marking glia^41^ or for Dlg marking photoreceptor axons^42^ (arrows in Fig. 7e, middle). While the exact distribution of carcinine was somewhat variable in these different backgrounds, its extracellular accumulation was consistently observed upon loss of Ncc69 function. This extracellular carcinine accumulation indicates that one of the consequences of loss of Ncc69 function is impaired carcinine uptake into photoreceptor axons.

To examine a possible contribution of this altered carcinine distribution to the *Ncc69* ERGs phenotype, we performed ERGs on *Ncc69*^r2^ flies fed histamine or carcinine. Loss of histidine decarboxylase (Hdc), the enzyme responsible for histamine neurotransmitter synthesis, blocks ON- and OFF transients, but these components can be restored with either histamine or carcinine feeding (Fig. 7f). Importantly, Ziegler *et al*.^41^ have shown that restoration of visual neurotransmission of *Hdc* mutants by histamine feeding requires Ebony, indicating supplemented histamine is shuttled through the same glia-dependent histamine-carcinine cycle as synaptically released histamine. Unlike *Hdc* mutants, dietary histamine or carcinine did not significantly restore ON- or OFF-transients in *Ncc69*^r2^ flies, consistent with an impairment in the glia-dependent histamine-carcinine cycle (Fig. 7f).

The extracellular accumulation of carcinine suggests that loss of *Ncc69* function may at least partially compromise CarT-mediated^25,35,36^ carcinine re-uptake into photoreceptor axons. This is unlikely to reflect a direct role of Ncc69 in carcinine transport into photoreceptor cells, given the *Ncc69* requirement in glia. Consistent with this notion, we could not detect any uptake of carcinine into Ncc69-expressing S2 cells (data not shown) using the same assay that demonstrated CarT-mediated carcinine transport^25,35,36^.

**Figure 8:**
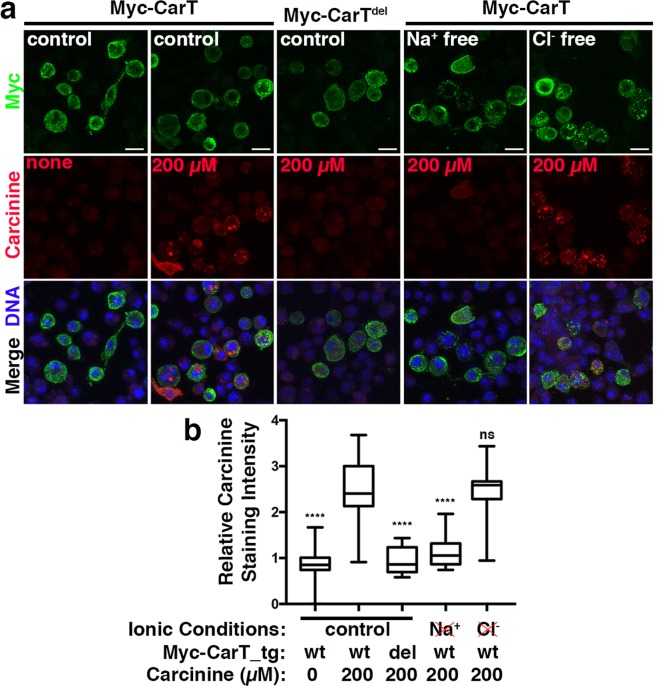
Carcinine uptake via CarT is dependent on Na^+^, but not Cl^−^ ions. (**a**) Micrographs of S2 cells transfected with the Myc-CarT transgene or, as negative control, the Myc-CarT^del^ transgene^25^ and cultured in control media supplemented with 0 or 200 µM carcinine or in media lacking Na^+^ or Cl^−^ supplemented with 200 µM carcinine as indicated. Staining for the Myc epitope, carcinine, and DNA indicate CarT-dependent carcinine uptake is dependent on Na^+^ but not Cl^−^ Ions. (**b**) Quantification of the carcinine signal normalized to the signal for the Myc epitope. ns, not significant; ****p < 0.0001 compared to Myc-CarT transfected cells in control ionic and carcinine-containing media.

CarT belongs to the Solute Linked Carrier 22 family of transporters, some of which operate as ion cotransporters^43^. To examine the ionic conditions necessary for CarT function we performed carcinine transport assays in the absence of Na^+^ or Cl^−^ ions. To minimize indirect effects of altered ionic environments on S2 cells, we limited their exposure to these conditions to one hour. These carcinine transport assays showed that extracellular Na^+^, but not Cl^−^, was necessary for carcinine uptake (Fig. 8). This finding suggested that an altered perisynaptic ionic environment in *Ncc69* lamina cartridges may contribute to the loss of visual neurotransmission in these mutants. Taken together, for the first time to our knowledge, we identify a role of glial *Ncc69* in visual neurotransmission and in possibly establishing the ionic environment necessary in the perisynaptic space to promote the histamine-carcinine cycle.

## Discussion

Na^+^-K^+^-2Cl^−^-cotransporters, like Ncc69, play a highly conserved role in regulating intracellular and extracellular ion concentrations in different cell types and tissues, ranging from kidneys to brains^3,9,10,12,13,16,44–46^. By utilizing the *Drosophila* visual system to further explore the role of Ncc69 in neuronal function, we discovered a novel role for glial Ncc69 in neurotransmission. Our data, coupled with observations in other systems^7,9,13,14^, indicate that, within the nervous system, distinct physiological consequences result from loss of Ncc69 function depending on cellular context. For example, glia-specific loss of Ncc69, despite being linked to an increased propensity of seizures^16^, did not affect action potential conduction or evoked post synaptic currents at larval neuromuscular junctions^13^. By contrast, our observations in the visual system indicate that glial loss of Ncc69 non-autonomously causes an almost complete loss of synaptic transmission to lamina neurons detected both behaviorally and via light-evoked ON- and OFF transients.

Changes in perisynaptic ion concentrations in the lamina are likely to have multiple consequences for the visual system. For example, excess extracellular K^+^ could depolarize the L1 and L2 laminar neurons, although the role of glial NKCC in perisynaptic K^+^ clearance has been controversial^46,47^. Alternatively, with altered extracellular Cl^−^ concentrations, the histamine-triggered influx of Cl^−^ into L1/2 lamina neurons^20^ could be compromised. In the extreme, altered Cl^−^ gradients across neuronal membranes can even reverse the response to neurotransmitters, such as histamine, that act on ligand-gated chloride channels, as has been observed for the responses to GABA during brain development^3^. Such effects on the lamina neurons may contribute to the slight increase in the size of their dendrites, but it is important to note that we functionally separated this effect from the loss of synaptic transmission. Ncc69 knockdown in neurons caused increased dendrite size without an effect on ON- and OFF-transients, whereas neurotransmission of *Ncc69* mutants, but not the size of L1/2 dendrites, was rescued by *Gliotactin*-Gal4-driven Ncc69 expression.

*Gliotactin*-Gal4 is expressed in perineurial and subperineurial glia which form the blood-brain barrier in *Drosophila*^30,48^. This is consistent with Ncc69, perhaps by raising Cl^−^ or lowering K^+^, contributing to the distinct ionic conditions of the peri-synaptic hemolymph as compared to the higher hemolymph K^+^ concentrations outside the nervous system. These effects on local ion concentrations may indirectly also affect neurotransmitter recycling or L1/2 lamina neuron activity.

Glial cells of the lamina also play a critical role in the recycling of histamine, the neurotransmitter of *Drosophila* photoreceptor neurons^19^. Histamine recycling is essential for normal vision since the rate of histamine synthesis is insufficient to sustain the high rate of synaptic release^49^. Synaptically released histamine is taken up by glia where Ebony^50^ catalyzes the condensation reaction between β-alanine and histamine to yield carcinine (Fig. 1b). Carcinine serves as a transport metabolite for histamine: it is released from glia, taken up by photoreceptors through the CarT transporter^25,35,36^ and finally recycled to histamine by Tan-mediated hydrolysis^51^. The accumulation of extracellular carcinine as a consequence of the loss of glial Ncc69 coupled with the failure of CarT to transport carcinine under Na^+^ free-conditions suggests that carcinine uptake into neurons may rely on perisynaptic ionic conditions maintained by Ncc69. Interestingly, CarT is a member of the SLC22 family of transporters and previous work has shown Na^+^-dependent transport activity for at least two other members, SLC22A4 and SLC22A5^43^.

At first glance the Na^+^-dependence of carcinine transport into photoreceptors may seem to predict increased carcinine clearance from the perisynaptic space in response to the loss of Ncc69-mediated Na^+^-import into glia. However, it is important to note that Ncc69 also transports K^+^ and Cl^−^ ions and is functionally coupled to the activity of other transporters, channels and the Na^+^/K^+^ ATPase^12,52^. Signaling between neurons and glia is bidirectional, and the alteration of intracellular Cl^−^ in glia through loss of Ncc69 could have secondary consequences on the release of glial transmitters that then influence neuronal behavior, in a carcinine-dependent or -independent manner^53^. Thus, the effect of loss of Ncc69 function on extracellular concentrations of ions are difficult to predict, and it is not clear whether the perisynaptic accumulation of carcinine is a direct or indirect consequence of altered ion conditions in the lamina. Furthermore, histamine distribution was not visibly altered in *Ncc69*^r2^ mutant heads, suggesting that the defect in carcinine uptake of *Ncc69* mutants may only be partial and not sufficient to explain the strong loss of visual neurotransmission.

The use of histamine as neurotransmitter in *Drosophila* is not restricted to the visual system, but has also been observed in peripheral mechanosensitive neurons^18^ that appear to also share specific histamine re-uptake mechanisms with photoreceptors^54^. It is not known whether Ncc69- or CarT-dependent mechanisms are involved in histamine recycling in these peripheral mechanoreceptors. This prompted our use of the automated visual startle assay, first described by Ni and colleagues^55^, to assess the behavioral consequences of the loss of Ncc69 in the visual system. This assay avoids any possible interference from altered mechanosensation that occurs during the physical handling of flies in a T-maze or during countercurrent assay. *Ncc69* mutant flies were unresponsive to the light pulse in the startle assay, similar to *CarT* mutants that are defective in histamine recycling. It is interesting, however, that this assay does not solely report on defects in compound eye signaling since *NorpA* flies, which lack significant visual responses from the compound eye due to the loss of phospholipase C activity^56^, still respond in the visual startle assay similar to wild-type flies^55^. This discrepancy suggests that the requirements for Ncc69 and CarT in this context are not restricted to neurotransmission from the canonical phototransduction pathway. Possibly, these transporters also contribute to histamine recycling and the functional output of the Rhodopsin7 and Cryptochrome-expressing pacemaker neurons in the central brain^55^. Furthermore, independently of the canonical PLC-beta encoded by *NorpA*, *PLC-21C* is required in light entrainment behavior mediated by Rh5 and Rh6 rhodopsins in R8 photoreceptor cells^57^. It is tempting to speculate that Ncc69-dependent synaptic transmission is required for one or more of these non-canonical phototransduction pathways leading to altered light-induced behavioral responses.

*Ncc69*-like loss of synaptic transmission was also observed when kinases WNK or Fray were depleted in glia cells, suggesting that this well-characterized phosphorylation cascade^15^ regulating Ncc69 activity is critical in visual glia for maintaining its function. Strikingly, we could rescue the loss of Drosophila WNK in glia cells with expression of any of the four mammalian WNKs. This suggests conservation of WNK function in regulating glial Ncc69. One possibility is that WNK acts as an ionic sensor^10,15,58^, to regulate Ncc69 and Cl^−^, K^+^ and Na^+^ in the lamina glia.

Our findings are the first to demonstrate a non-autonomous role for glial NKCC and the regulatory kinases WNK and Fray in synaptic transmission. Given the conservation of Ncc69 and its regulatory kinase cascade, and the observed rescue of Drosophila WNK depletion by the mammalian WNKs, it will be interesting to see whether such a glial role for NKCC transporters in neurotransmission and neurotransmitter recycling is conserved in the mammalian brain.

## Methods

### Fly work

Flies were maintained using standard conditions. Fly lines *repo-*Gal4 (BDSC_7415), longGMR-Gal4 (BDSC_8121), *elav*-Gal4 (BDSC_8760), *Hdc*^MB07212^ (BDSC_25260), R10D10-Gal4 (BDSC_69558), R32H04-Gal4 (BDSC_49734), R29A12-Gal4 (BDSC_49478), and R19C02-Gal4 (BDSC_49282) were provided by the Bloomington *Drosophila* Stock Center. The mCD8-GFP and 3XPax3-GFP markers and the *carT*^43A^ mutant have been described^25^. UAS-WNK-RNAi and UAS-fray-RNAi transgenes and their effectiveness in knocking down the relevant target have been described^10^. The UAS-Ncc69-RNAi line (VDRC KK106499) was obtained from the Vienna *Drosophila* Resource Center (VDRC). The *Ncc69*^r1^*, Ncc69*^r2^ and *Gli*-Gal4; UAS-Ncc69 flies^13^ were a gift from Dr. William Leiserson (Yale University, New Haven, CT). The MZ709-Gal4 line was a gift from Dr. Hong-Sheng Li (University of Massachusetts Medical Center, Worcester, MA).

### Molecular Biology

All UAS-mammalian WNK transgenic lines were generated using the Gateway cloning method (ThermoFisher) with Platinum Pfx DNA polymerase (ThermoFisher 11708013), pENTR/D-TOPO Cloning Kit (ThermoFisher, K240020), LR Clonase II (ThermoFisher, 11791020), and the Gateway compatible destination vector pUASg.attB, obtained from Johannes Bischof and Konrad Basler (Zurich, Switzerland)^59^. Template vectors (pROSA-rWNK1.3, pCMV7.1-hWNK2, pCMV7.1-hWNK3.2 and pCMV5-mWNK4) were obtained from Chou-Long Huang (UT Southwestern, Dallas, TX). Mammalian WNK PCR amplicons were gel purified and incorporated into the attL containing entry vector pENTR via directional TOPO cloning per manufacturer’s protocol.

WNK1 primers used were 5′ CACCATGTCTGACGGCACCGCAGAG 3′ and 5′ GGTGGTCCGTAGGTTGGAAC 3′.

WNK2 primers used were 5′ CACCATGGACGGCGATGGCGGCCGCCGAG 3′ and 5′ GTCAGGCTTCTCACTCTCAGGATCTGG 3′.

WNK3 primers used were 5′ CACCATGGCCACTGATTCAGGGGATCCAGC 3′ and 5′ TTTAGGACCAGGAGGGATTGTGGCAGG 3′.

WNK4 primers used were 5′ CACCATGCTAGCACCTCGAAATACGGAGACTGG 3′ and 5′ CATCCTGCCAATATCCCCGGCGAATG 3′.

Full-length pENTR-WNK clones were then shuttled into the attR containing destination vector pUASg.attB by LR clonase reactions and sequences confirmed. Midiprep DNA was sent to Rainbow Transgenic Flies for microinjection into stock line #24483 (*M[vas-int.D]ZH-2A, M[3xP3-RFP.attP]ZH-51D*). Stocks for each transgenic line were generated from single male transformants, and confirmation of the UAS-transgenes was performed by sequence-specific primers. All transgenic *WNK* lines were outcrossed for 5 generations to the Rodan laboratory *wBerlin* genetic background.

### Electroretinogram recordings

ERGs were recorded as previously described^25^. In brief, voltage measurements of immobilized female flies were recorded with electrodes containing 2 M NaCl placed on the corneal surface and inserted into the thorax. Measurements were filtered through an electrometer (IE-210; Warner Instruments), digitized with a Digidata 1440 A and MiniDigi 1B system (Molecular Devices), and recorded using Clampex 10.2 (Axon Instruments). Light pulses (1 s at 600 lux, unless otherwise noted) were computer controlled (MC1500; Schott). Five ERG recordings from at least ten flies were performed in triplicate and quantified with Clampfit software (Axon Instruments). Light intensities were measured using a Fisher Scientific Dual-Range Light Meter (Fisher scientific).

### Light-startle behavior assay

The assay was adapted from a previously described method^55^. Flies were collected zero to one day post-eclosion and reared under a standard LD cycle for 3 days. 16 flies per genotype were placed into individual Drosophila Assay Monitoring (DAM) chambers (TriKinetics Inc, Waltham, MA). The DAM monitors were placed into a dark incubator at ZT4 for a 2-h dark adaptation period followed by a 5-min pulse of 500-lux light at ZT6. Fly motor activity was automatically recorded with a DAMSystem3.0 and DAMFileScan11.0 (TriKinetics Inc). Raw data were exported to Microsoft Excel and processed in GraphPad Prism. The change in activity following the light pulse was calculated as [*mean beam breaks for 10 min. post-pulse*] – [*mean beam breaks for 10 min. pre-pulse*]. Three independent technical replicas of 16 different flies each per genotype were performed on separate days. Dead flies and rare hyperactive outliers (greater than 3 standard deviations) were removed before final statistical analysis.

### Histology

Fly heads were dissected in hemolymph-like solution^60^ to remove the proboscis and posterior cuticle, fixed either for 1 hour in 4% paraformaldehyde or 4 h in ice-cold 4% 1-ethyl-3-(-3-dimethylaminopropyl) carbodiimide (wt/vol, Sigma) in 0.1 M phosphate buffer solution, washed overnight in 25% (wt/vol) sucrose in phosphate buffer (pH 7.4), embedded in optimal cutting temperature compound, frozen in dry ice, and sectioned at 20-μm thickness on a cryostat microtome (Leica). Sections were incubated overnight with antibodies to histamine (1:1000, Sigma, cat# H7403 pre-absorbed with 200 µM carcinine) or carcinine (1:1000, Immunostar, cat# 22939 pre-absorbed with 200 µM histamine). Specificity of these stainings for histamine and carcinine was confirmed in *ebony* and *tan* mutants as described^25^. Other antibodies used include anti-Ebony (gift from Bernhard Hovemann^39^), anti-Black (gift from Bernhard Hovemann^39^), anti-Bruchpilot (nc82, Hybridoma Bank), anti-NCC69 (gift from Jim Turner, NIH)^11^, anti-DLG (4F3, Hybridoma Bank), and anti-GFP (GFP-1020, Aves Labs). Secondary antibodies were labeled with Alexa488 (1:500, Molecular Probes, cat# A-11008), Alexa568 (1:500, Molecular Probes, cat# A-11011), or Alexa647 (1:500, Molecular Probes A-21235). Where indicated, Topro-3 Iodide (Molecular Probes, T3605) was used to stain DNA. Images were captured using a Zeiss LSM510 confocal microscope with a 20 × NA 0.75 or a 63 × NA 1.4 lens on an inverted confocal microscope (LSM510 Meta; Carl Zeiss) at 21–23 °C or a 63 × NA 1.4 objective with Airyscan detector (LSM880, Airyscan; Carl Zeiss) at 23 °C. Images were processed in Zen Blue (Carl Zeiss) and ImageJ (NIH).

### Electron microscopy

After removal of the proboscis and posterior cuticle, fly heads were fixed overnight at 4 °C in 2% paraformaldehyde, 2% glutaraldehyde in 0.1 M phosphate buffer, pH 7.4. Heads were washed three times in 0.1 M phosphate buffer, followed by 3 washes in 0.1 M sodium cacodylate buffer. Fixed heads were embedded in 3% agarose, tissue samples were then rinsed in 0.1 M sodium cacodylate buffer and post-fixed in 1% osmium tetroxide and 0.8% potassium ferricyanide in 0.1 M sodium cacodylate buffer for 90 min at room temperature. After three rinses in water, they were en-bloc stained with 4% uranyl acetate in 50% ethanol for two hours, dehydrated with increasing concentrations of ethanol, transitioned into resin with propylene oxide, infiltrated with Embed-812 resin, and polymerized at 60 °C overnight. 550 nm sections were cut and stained with toluidine blue to confirm orientation and section depth. Cartridges sizes and L1/2 areas were measured from such thick sections for Fig. 5c. Some blocks were then thin sectioned at 70 nm with a diamond knife (Diatome) on a Leica Ultracut 6 ultramicrotome (Leica Microsystems) and collected onto formvar-coated, glow-discharged copper grids, post-stained with 2% aqueous uranyl acetate and lead citrate. Images were acquired on a Tecnai G^2^ spirit transmission electron microscope (FEI) equipped with a LaB_6_ source using a voltage of 120 kV. From such images cartridges sizes and L1/2 areas were measured for Fig. 2c.

### Carcinine uptake experiments

Drosophila S2 cells were plated and transfected with pMT-Myc-CarT or the pMT-Myc-CarT^del^ as described^25^. Post 24-hour induction with CuSO_4_, cells were washed twice in either modified Schneider’s, Na^+^ free, or Cl^−^ free media (Supplemental Table 1) as indicated and incubated with the respective media containing 0 µM or 200 µM carcinine for 1 hour. Cells were then transferred to ice and fixed with 4% ethyl-3-(-3-dimethylaminopropyl) carbodiimide (wt/vol, Sigma) in ice-cold 0.1 M phosphate buffer solution. Fixed cells were stained with Myc and carcinine antibodies as described^25^. Quantification was performed in ImageJ by normalizing the integrated density of the carcinine signal by that of the Myc signal. Media isotonicity was measured via a Vapro pressure osmometer (model 5520, Wescor).

### Image Quantification

Cartridge area and combined L1 and L2 area measurements were obtained using Macnification software (Orbicule). Based on morphology, cartridge area was defined by the region contained within the outer most boundaries of the R1-6 photoreceptors axons. L1 and L2 area was identified by its central location within a cartridge and the lack of synaptic vesicles. Only cartridges with distinct morphological boundaries were analyzed, and for each genotype we analyzed at least 50 cartridges in two replicate experiments.

To quantify carcinine levels within the lamina, confocal stacks were imported to ImageJ (National Institutes of Health) and masks of the lamina region generated based on *repo*-Gal4 driven expression of UAS-mCD8::GFP. Integrated pixel intensities of carcinine immunoreactivity per unit area were determined from at least ten individual lamina per genotype.

### Statistics

Statistical significance was determined using GraphPad Prism 6 to perform one-way ANOVA, followed by Tukey’s or Bonferroni’s for multiple comparisons or two-tailed Students t-test for pair-wise comparisons.

## Supplementary information

Supplementary Information
