# Supplementary material for: The glial sodium-potassium-2-chloride cotransporter is required for synaptic transmission in the Drosophila visual system
Source: Sci Rep. 2019 Feb 21;9:2475. doi: 10.1038/s41598-019-38850-x (PMC6385505; doi:10.1038/s41598-019-38850-x)
Supplement: Supplementary file 1 — Supplementary Information [file 41598_2019_38850_MOESM1_ESM.pdf]

Supplemental Information:

The glial sodium-potassium-2-chloride cotransporter is required for synaptic transmission in the *Drosophila* visual system.

Drew Stenesen*[,1,6], Andrew T. Moehlman*[,1], Jeffrey N. Schellinger[2], Aylin R. Rodan[2,4,5†] and Helmut Krämer[1,3†]

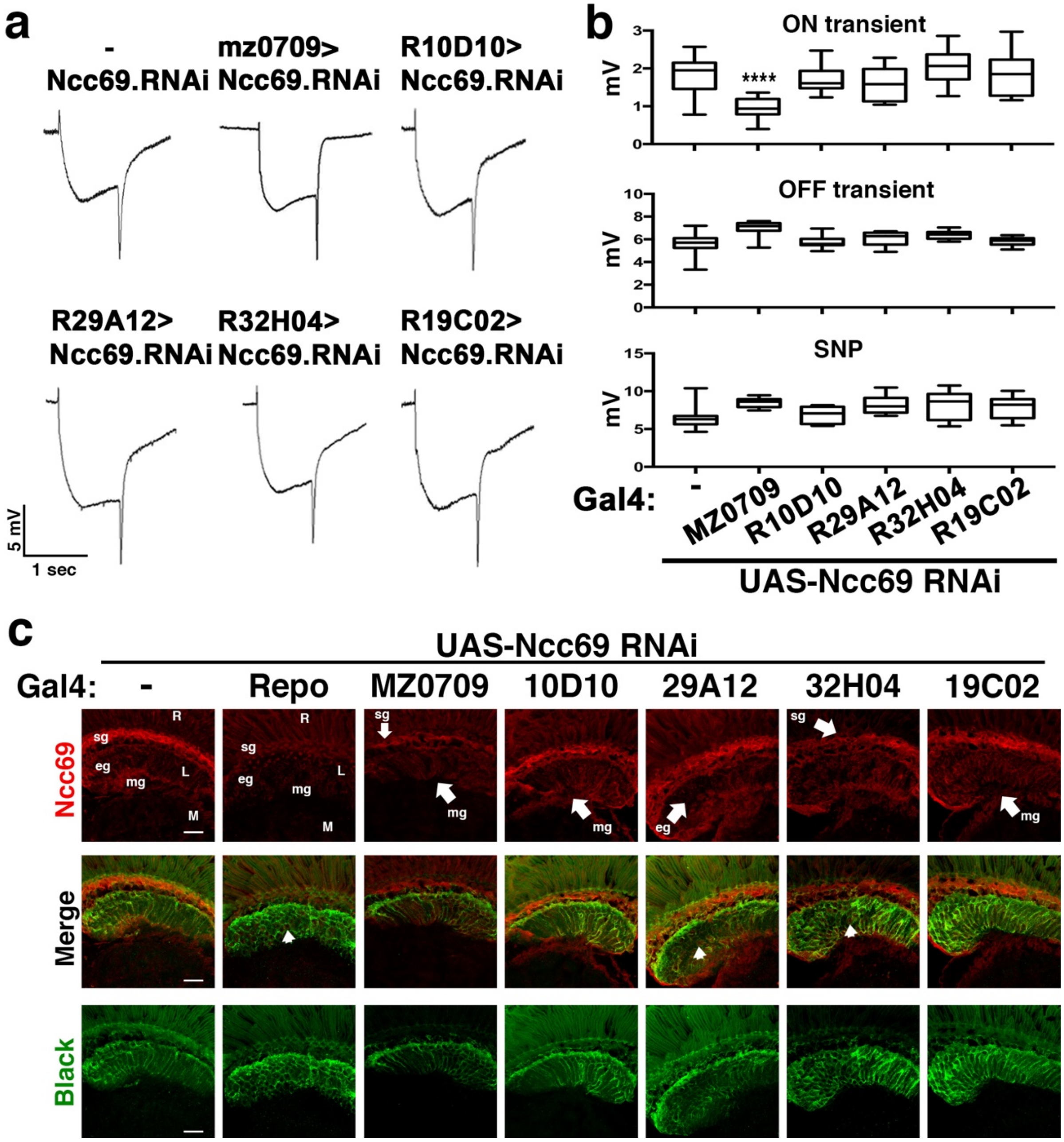

Supplemental Figure S1. Knockdown of Ncc69 in individual glia subcompartments does not recapitulate knockdown in all glia. (**a**) Representative ERG traces from flies with Ncc69-RNAi crossed to the following genotypes: OreR (control), MZ0709 (satellite glia and marginal glia), *R10D10-Gal4* (distal satellite glia and marginal glia), *R29A12-Gal4* (epithelial glia), *R32H04-Gal4* (proximal satellite glia and some epithelial glia), and *R19C02-Gal4* (marginal glia). (**b**) Quantifications of ON and OFF transients as well as sustained negative potentials from at least 10 flies per genotype, from 2 independent experiments. Lines indicate means, boxes show 25th-75th quartiles and whiskers show min and max. (**c**) Representative projected images for Ncc69 (red) and Black (epithelial glia marker, green) from indicated genotypes. R: retina, L: lamina, M: medulla, sg: satellite glia, mg: marginal glia, eg: epithelial glia. Images acquired on LSM880, 63X objective. Scale bar: 20 μM.

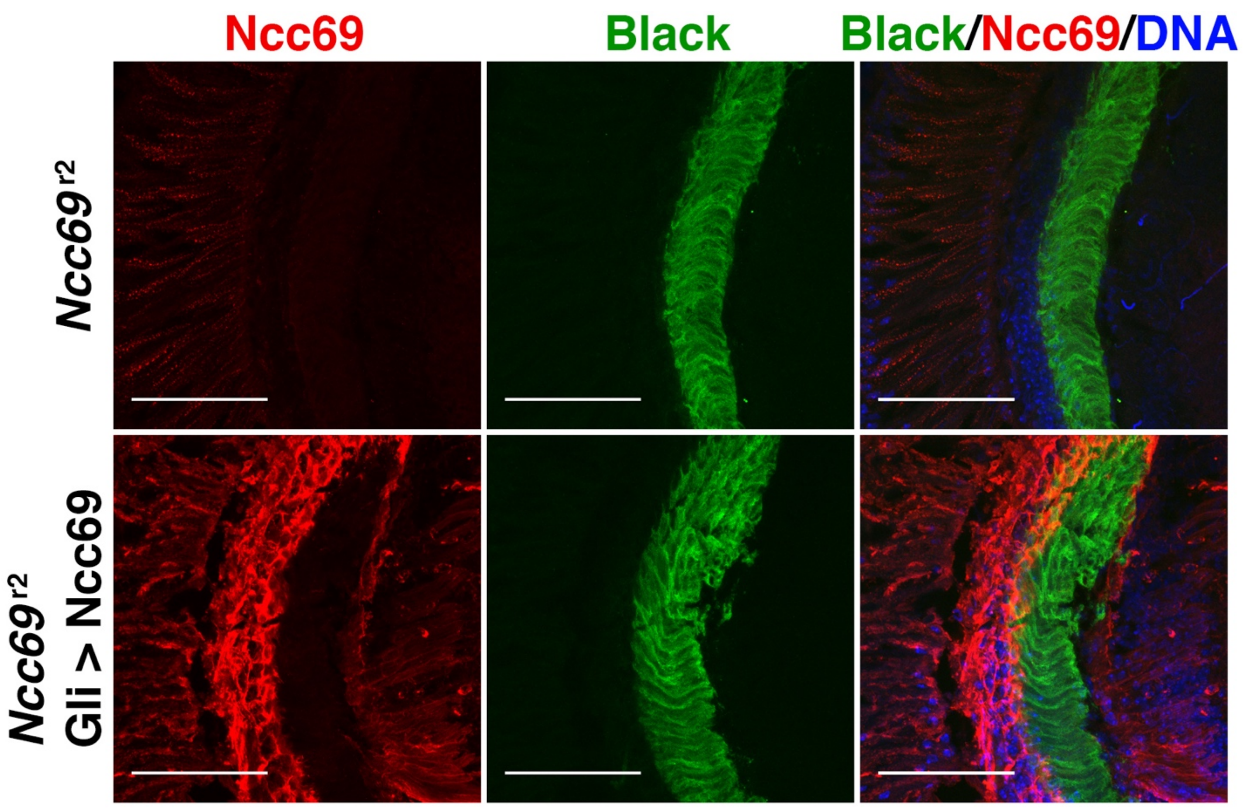

Supplemental Figure S2. Expression of *Gli*-Gal4, UAS-Ncc69-HA in lamina glia. Representative immunostaining for Ncc69 (red) and Black (epithelial glia, green) in sections from *Ncc69*^{R2} or *Gli*-Gal4, UAS-Ncc69.HA*; Ncc69*^{R2} flies. *Gli*-Gal4-driven expression of Ncc69 is highly enriched in satellite glia. Images acquired on LSM880, 63X objective. Scale bar: 50 µM.

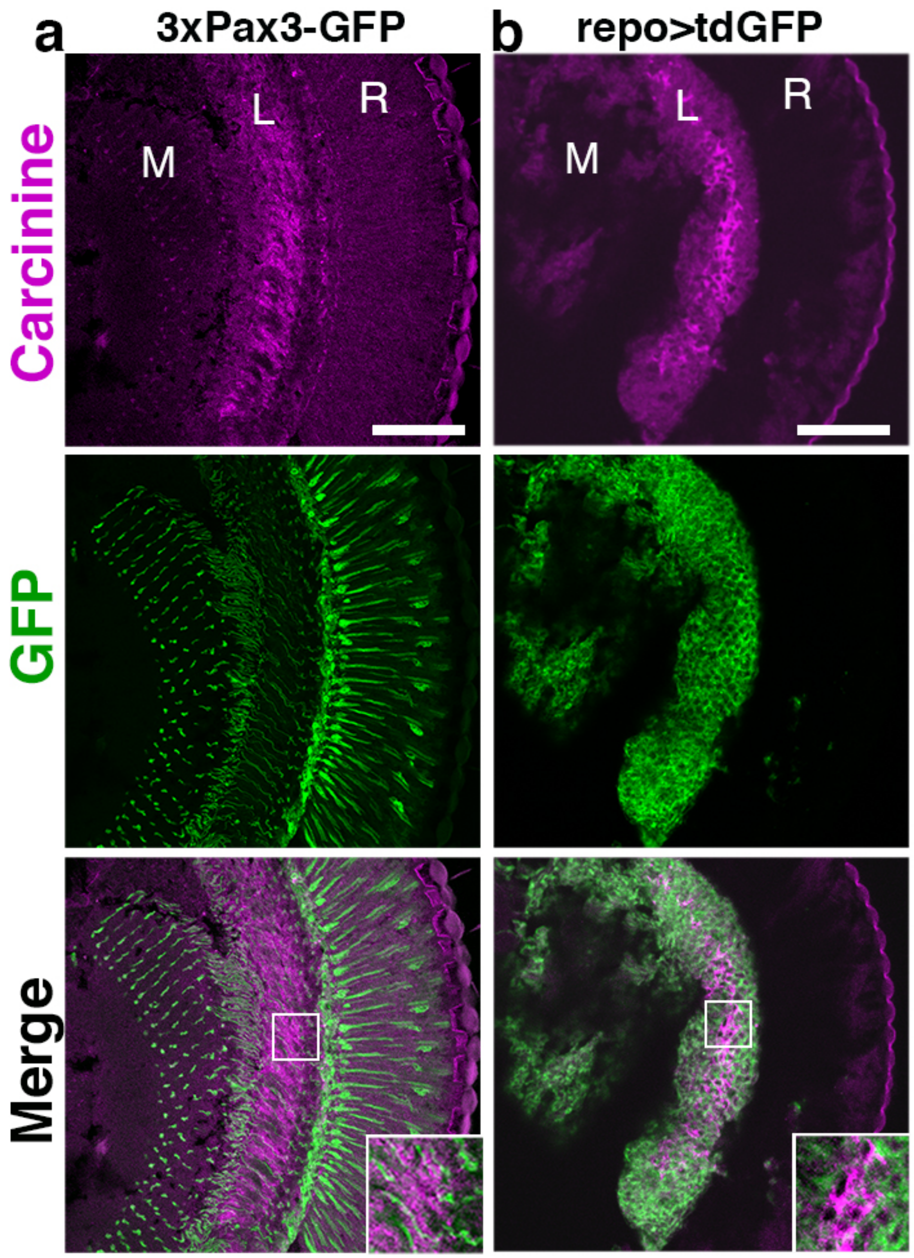

Supplemental Figure S3. Carcinine accumulation after glial Ncc69 knockdown does not colocalize with cytosolic GFP in either photoreceptors or glia. Micrographs of sections stained for carcinine from fly heads expressing the *repo*-Gal4-driven UAS-Ncc69-RNAi and cytoplasmic glial tdGFP (**b**), or with photoreceptor-specific cytosolic 3xPax3-GFP (**a**). Low levels of colocalization (see insets) indicate that carcinine was trapped extracellularly. Scale bars: 50 μm. M: medulla, L: Lamina, R: Retina.
